# A Case of Severe Prekallikrein Deficiency Manifesting as Isolated Activated Partial Thromboplastin Time (aPTT) Prolongation

**DOI:** 10.7759/cureus.79315

**Published:** 2025-02-19

**Authors:** Divya Viswanathan, Abiram Sivanandam, Vincent Yeung

**Affiliations:** 1 Internal Medicine, Rutgers University New Jersey Medical School, Newark, USA; 2 Hematology and Oncology, Rutgers Cancer Institute of New Jersey, New Brunswick, USA

**Keywords:** coagulation, hematology, prekallikrein, thrombosis, transfusion

## Abstract

We present the case of a 54-year-old female who was admitted to the medicine service for a left foot ulcer with cellulitis requiring hallux amputation. Pre-surgical laboratory findings were significant for an isolated elevation in activated partial thromboplastin time (aPTT). Hematology was consulted for evaluation of prolonged aPTT prior to surgery given potential for bleeding risk. Further laboratory studies obtained were significant for normal factor levels, negative hypercoagulable workup, normal kininogen activity, and severe prekallikrein deficiency. The patient was pre-operatively given one unit of fresh frozen plasma (FFP). She had minimal blood loss during her procedure with no additional blood products required post intervention. Because patients with prekallikrein deficiency have preserved auto-activation of factor XII, they rarely manifest with increased bleeding risk or thrombotic risk. Thus, presurgical optimization of patients with severe prekallikrein deficiency should be standardized to limit delay of care. Our review suggests that routine prophylactic administration of blood products for pre-operative management of this condition is not indicated but may be considered if the patient is at high risk of hemorrhage or clinical deterioration. Thrombotic risk should also be considered in these patients.

## Introduction

Prekallikrein (PK), otherwise known as Fletcher factor, acts as the precursor to kallikrein, a serine protease that is responsible for the initiation of the intrinsic pathway of the coagulation cascade and promotes factor XII activation [1]. PK deficiency is thought to be caused by mutations in the KLKB1 gene, which is located on chromosome 4q35 and inherited in an autosomal recessive pattern [2,3]. This is a rare disorder in which patients often present with prolonged activated partial thromboplastin time (aPTT) secondary to slower activation of factor XII. Factor XII is able to autoactivate without the presence of kallikrein [1]. Due to continued formation of this factor’s active phase, patients often do not have an associated increased risk of bleeding, as the coagulation cascade remains intact [1]. 

## Case presentation

A 54-year-old female with a past medical history of hypertension, left foot ulcer, and type 2 diabetes complicated by neuropathy, glaucoma, and bilateral cataracts status post intraocular lens placement presented to the emergency department in June 2024 with left foot pain, swelling and chills. X-ray of the left foot showed soft tissue swelling of the first toe with ulceration and air present. She was started on empiric cefepime and metronidazole for prior wound cultures in May 2024 growing group B streptococcus and Morganella morganii. Initial labs seen in Table 1 were significant for an isolated elevation of PTT to 107.7. The patient notably was not on any anticoagulation prior or during hospitalization. The patient denied any prior history of known bleeding disorder, episodes of spontaneous bleeding, or known prior abnormalities in blood work. Podiatry evaluated the patient and recommended left foot debridement with possible hallux amputation, but requested further investigation of elevated PTT prior to surgery. 

**Table 1:** Initial laboratory studies BUN: blood urea nitrogen; GFR: glomerular filtration rate; WBC: white blood count; INR: international normalized ratio; aPTT: activated partial thromboplastin time; ESR: erythrocyte sedimentation rate; CRP: c-reactive protein

Parameter	Lab value	Reference range	Units
Glucose	237	70-109	mg/dL
BUN	17	6-20	mg/dL
Creatinine	0.8	0.5-1.0	mg/dL
Sodium	143	133-145	meq/L
Potassium	4.8	3.5-4.8	meq/L
Chloride	109	97-110	meq/L
Bicarbonate	24	23-30	meq/L
Calcium	9.7	8.4-10.2	mg/dL
GFR	88	>60	mL/min
WBC	7.0	4.0-11.0	x 10^3/muL
Hemoglobin	12.9	12.0-16.0	g/dL
Platelets	333	150-450	x 10^3/muL
Protime	13.3	12.1-14.8	seconds
INR	1.0	0.9-1.17	N/A
aPTT	107.7	24.0-34.2	seconds
ESR	90	0-20	mm/hour
CRP	107	0-5	mg/L
Hemoglobin A1c	13.4	4.8-5.9	%

Hematology was consulted due to findings of isolated elevation in PTT for pre-operative assessment. A mixing study was performed and showed correction sustained at 0 minutes, 30 minutes, 60 minutes, and 90 minutes, consistent with factor deficiency. The patient’s factor levels and further laboratory values are further detailed in Table 2. These studies were notable for normal factor levels (VIII, IX, XI, XII), normal von Willebrand factor (VWF) activity, negative testing for lupus anticoagulant, negative anticardiolipin antibodies and beta-2 glycoprotein antibodies. Prekallikrein levels were obtained but the result did not return until after the patient's hospitalization. The patient was transfused one unit of fresh frozen plasma (FFP) pre-operatively due to an unclear etiology of prolonged aPTT prior to foot surgery. The patient's aPTT corrected to 34.2 seconds after the FFP transfusion. Patient underwent partial hallux amputation with no major bleeding complications with around 10 milliliters of blood lost during the procedure. The patient did not require any additional blood products intra or post-operatively with no clinical signs of bleeding or thrombosis following amputation. During a follow-up appointment with hematology as an outpatient, the patient was noted to have severe prekallikrein deficiency with activity measured as <5, normal kininogen activity, and elevated aPTT to 90.9 seconds. 

**Table 2:** Additional laboratory studies VWF: von-Willebrand factor; SPEP: serum protein electrophoresis; B2: Beta-2

Parameter	Lab value	Reference range	Units
Factor VIII	286	50-166	%
Factor IX	188	60-176	%
Factor XI	167	54-198	%
Factor XII	91	45 - 183	%
VWF antigen	143	50-160	%
VWF activity	56	50-200	%
Dilute Russell’s Viper Venom Test (DRVVT)	Negative	N/A	N/A
B2-glycoprotein IgG	<9	0-20	GPI IgG Units
B2-glycoprotein IgA	<9	0-25	GPI IgA Units
B2-glycoprotein IgM	<9	0-32	GPI IgM Units
Anticardiolipin IgG	<9	0-14	GPL U/mL
Anticardiolipin IgM	<9	0-12	GPL U/mL
SPEP	No M-spike observed	N/A	N/A
Serum Free Kappa:Lambda Ratio (SFKLR)	1.89	0.26 - 1.65	N/A
Kininogen	115	65-135	%
Prekallikrein	<5	>=50	%

## Discussion

The kallikrein-kinin system is a cascade that is involved in stimulation of coagulation as well as the formation of bradykinin and its related peptides which mediate processes such as inflammation and vascular permeability [4]. Factor XII is initially converted to factor XIIa secondary to a process known as contact activation. This autoactivation occurs upon the protein’s exposure to negatively charged surfaces [4]. In its active state, factor XIIa activates factor XI and aids the subsequent conversion of prekallikrein to kallikrein [4]. Plasma kallikrein can in turn mediate conversion of factor XII to its active state at a rate faster than its own autoactivation [4]. Patients lacking prekallikrein therefore are unable to undergo this faster activation of factor XII, but have preserved function of factor XII autoactivation, keeping the coagulation cascade intact.

Kallikrein also has the additional role of conversion of plasminogen to plasmin, serving as a feedback mechanism to enhance the fibrinolytic response to maintain clotting regulation [5]. It has also been noted that plasmin reciprocally may play a part in the activation of factor XII, and therefore the activation of prekallikrein into kallikrein [6]. Due to these factors, PK deficiency often does not manifest with symptoms and is often incidentally observed as an isolated elevation of PTT. However, there have been few reported cases of prekallikrein deficiency manifesting with symptoms of hemorrhage or thrombosis.

A previous study detailed 111 cases of severe PK deficiency from 89 families [2]. Of these cases, only four major bleeding events were described amongst 96 patients during their lifetimes and included spontaneous hematemesis, intracranial hemorrhage following cerebral vein thrombosis, prolonged bleeding after tonsillectomy, and intracranial hemorrhage in a premature newborn [2]. Similarly, long-term outcomes of 53 patients with severe PK deficiency were studied [7]. Of those 53 patients, 20 underwent surgical procedures without prophylactic blood products, and 16 had no acute bleeding events perioperatively [7]. Other reported cases include recurrent spontaneous cerebrovascular accident (CVA) in a 32-year-old female and recurrent mucosal hemorrhage in a 75-year-old female [8,9]. 

Because prekallikrein deficiency will affect the measurement of PTT, in patients with indication for anticoagulation, anti-Xa levels should be routinely monitored [10]. Anti-Xa is able to measure the effect of heparin by measuring the inhibition of factor Xa. While PTT measures the activity of the intrinsic pathway, which is affected by decreased levels of prekallikrein, factor Xa is unaffected in this process. 

Patients with PK deficiency have also been reported to have thrombotic events, both venous and arterial. Four siblings with severe PK deficiency were previously studied, three of whom presented with venous thrombosis, and one with acute stroke [11]. A similar study describes 75 patients with PK deficiency, nine presenting with thrombotic events but only two without other predisposing factors [12]. Animal models comparing factor XII and PK deficiencies suggest that factor XII has a greater effect in thrombus formation and that PK plays a more supportive role [13]. Despite the fact that PK deficiency is an inherited clotting cascade disorder, the condition itself may not be protective against thrombosis. Because PK has a role in activating plasminogen to form plasmin and enhance fibrinolysis, PK deficiency may paradoxically lead to thrombotic events, as seen in these cases [5]. 

Overall, the low incidence of hemorrhagic and thrombotic events in these patients further provides evidence that routine administration of blood products or pro-hemostatic products prior to surgery is not routinely indicated unless there is a high clinical suspicion for a potential hemorrhagic event. Patients with issues in coagulation predisposing them to thrombotic events may warrant closer monitoring post intervention.

## Conclusions

Prekallikrein deficiency commonly presents as isolated PTT elevation without clinically significant bleeding events. Given that these patients are mostly identified prior to surgical intervention, it is important to establish consistent management guidelines for these patients so as to avoid delays in care. Patients with PK deficiency are at low risk of developing bleeding events throughout their lifetimes. From our review, there is no consistent data supporting the use of prophylactic blood products pre-operatively in these patients. Interestingly, these patients appear to be at similar risk of thrombosis as unaffected individuals despite their coagulation defect. In patients with other factors predisposing them to bleeding the use of blood products may be warranted. Patients with risk of thrombosis may need to be monitored for signs of potential thrombotic events. 
